# Ultrasound Elastography in Evaluation of Peripheral Pulmonary Lesions

**DOI:** 10.15388/Amed.2026.33.1.7

**Published:** 2026-07-22

**Authors:** Abdul Rouf, Uma Debi, Shritik Devkota, Mandeep Garg, Navneet Singh, Amanjit Bal, Harish Bhujade, K. T. Prasad

**Affiliations:** 1 Department of Radiodiagnosis and Imaging, Post Graduate Institute of Medical Education & Research, Chandigarh, India; 2 Department of Pulmonary Medicine, Post Graduate Institute of Medical Education & Research, Chandigarh, India; 3 Department of Histopathology, Post Graduate Institute of Medical Education & Research, Chandigarh, India

**Keywords:** ultrasound elastography, SWE, elastography, lung lesions, ultragarso elastografija, elastografija, plaučių pažeidimai

## Abstract

**Background:**

Differentiating benign from malignant peripheral pulmonary lesions (PPLs) remains a clinical challenge, especially in resource-limited settings. While computed tomography is the standard imaging modality, it involves radiation exposure and often lacks specificity. *Shear Wave Elastography* (SWE) is a non-invasive ultrasound technique that quantifies tissue stiffness and may aid in the evaluation of PPLs.

**Methods:**

In this prospective observational study, 42 patients with peripheral pulmonary lesions underwent transthoracic SWE prior to histopathological evaluation. Elastography values were compared between benign and malignant lesions. Statistical analysis included ROC curve assessment, logistic regression, and correlation with histopathological and microbiological outcomes.

**Results:**

Of the 42 patients, 64.3% had malignant and 35.7% had benign lesions. The mean SWE value for malignant lesions was significantly higher (6.82 ± 2.27 kPa) than for benign lesions (3.72 ± 2.23 kPa; *p* <0.001). The AUROC for mean SWE values in predicting malignancy was 0.849 (95% CI: 0.713–0.986), demonstrating good diagnostic performance with statistically significant difference (*p* = <0.001), with a cutoff of ≥4.8 kPa yielding 85% sensitivity and 80% specificity. Squamous cell carcinoma exhibited the highest stiffness among malignancies. Certain benign lesions, particularly tuberculosis, showed elevated stiffness, resulting in false positives. Logistic regression identified mean SWE as an independent predictor of malignancy (OR = 2.11, *p* = 0.030).

**Conclusion:**

Transthoracic SWE is a non-invasive, radiation free and promising tool for evaluating PPLs, offering good diagnostic accuracy in distinguishing malignant from benign lesions. It holds particular promise in settings where access to advanced imaging or biopsy is limited, and may assist in triaging patients for early tissue diagnosis.

## Abstract (it)

**Įvadas:**

Atskirti nepiktybinius ir piktybinius periferinius plaučių pažeidimus (PPL) tebėra klinikinis iššūkis, ypač ten, kur trūksta išteklių. Nors kompiuterinė tomografija yra standartinis vaizdinimo metodas, ji susijusi su radiacijos poveikiu ir dažnai nėra pakankamai specifiška. Šlyties bangų elastografija (SWE) yra neinvazinis ultragarso metodas, leidžiantis kiekybiškai įvertinti audinių standumą ir galintis padėti vertinant PPL.

**Metodai:**

Šiame perspektyviajame stebėjimo tyrime 42 pacientams su periferiniais plaučių pažeidimais prieš histopatologinį vertinimą buvo atlikta transtorakalinė 2D-SWE. Buvo lyginami gerybinių ir piktybinių pažeidimų elastografijos rodikliai. Statistinė analizė apėmė ROC kreivės vertinimą, logistinę regresiją ir koreliaciją su histopatologiniais bei mikrobiologiniais rezultatais.

**Rezultatai:**

Iš 42 pacientų 64,3 % turėjo piktybinių, o 35,7 % – gerybinių pažeidimų. Vidutinė SWE vertė piktybinių pažeidimų buvo statistiškai reikšmingai didesnė (6,82 ± 2,27 kPa) nei gerybinių pažeidimų (3,72 ± 2,23 kPa; p < 0,001). Vidutinių SWE verčių AUROC piktybiniam navikui prognozuoti buvo 0,849 (95 % CI: 0,713–0,986), o tai rodo gerą diagnostinį našumą su statistiškai reikšmingu skirtumu (p = <0,001), kai riba ≥ 4,8 kPa suteikia 85 % jautrumą ir 80 % specifiškumą. Plokščialąstelinė karcinoma pasižymėjo didžiausiu standumu tarp piktybinių navikų. Tam tikros gerybinės pažeidimų rūšys, ypač tuberkuliozė, pasižymėjo padidėjusiu standumu, dėl ko buvo gauti klaidingi teigiami rezultatai. Logistinė regresija nustatė, kad vidutinė SWE yra nepriklausomas piktybinių navikų prognozės rodiklis (OR = 2,11, p = 0,030). Nebuvo užfiksuota jokių su procedūra susijusių komplikacijų.

**Išvada:**

Transtorakalinė 2D-SWE yra saugi, greita ir veiksminga PPL vertinimo priemonė, užtikrinanti gerą diagnostinį tikslumą atskiriant piktybinius pažeidimus nuo gerybinių pažeidimų. Ji yra ypač perspektyvi tomis aplinkybėmis, kur prieiga prie pažangių vaizdinimo metodų ar biopsijos yra ribota, ir gali padėti atrinkti pacientus ankstyvajai audinių diagnozei.

## Introduction

Lung lesions, including benign and malignant types, encompass a wide spectrum of pathological conditions, with lung cancer being the most significant due to its high global incidence and mortality rates. Lung cancer remains one of the leading causes of cancer-related morbidity and mortality worldwide, accounting for a substantial proportion of oncologic deaths each year [1]. Early and accurate diagnosis of peripheral pulmonary lesions (PPLs), particularly malignant ones, is critical to improving prognosis and guiding treatment strategies.

Traditionally, *Computed Tomography* (CT) has been the imaging modality of choice for evaluating lung lesions due to its high spatial resolution and detailed anatomical imaging [2]. However, CT involves ionizing radiation exposure and may not always be readily available for bedside assessments or repeated follow-ups, especially in vulnerable populations such as children, pregnant women, or critically ill patients.

*Ultrasound* (US) has emerged as a valuable adjunct imaging modality in thoracic evaluation, particularly for peripheral lung lesions that are in contact with the pleura. As a radiation-free, real-time, and portable tool, ultrasound offers advantages in point-of-care settings, bedside examinations, and interventional procedures. It is cost-effective, widely available, and facilitates dynamic assessment of lesions and surrounding structures [3].

*Ultrasound Shear Wave Elastography* (US SWE) is an advanced, non-invasive imaging technique that quantitatively assesses tissue elasticity by measuring the velocity of mechanically induced shear waves propagating through tissue. It provides real-time information on tissue stiffness, which can aid in differentiating benign from malignant lesions. Malignant tumors generally exhibit increased stiffness due to their dense cellularity, desmoplastic stroma, and higher collagen content, whereas benign lesions tend to be softer [4]. US SWE has demonstrated diagnostic utility in evaluating various organs such as the liver, breast, thyroid, prostate, and lymph nodes [5].

Despite its established role in other organ systems, the application of elastography in lung imaging has been relatively limited due to the acoustic challenges posed by air-filled alveoli and associated artifacts. However, with recent technological advancements, there is a growing body of evidence supporting the feasibility and potential utility of US elastography in the assessment of peripheral pulmonary lesions [6,7].

In this study, we aimed to evaluate the role of ultrasound shear wave elastography in differentiating between benign and malignant peripheral lung lesions by determining and correlating elasticity values with histopathological/microbiological findings.

## Materials and Methods

### Study Design and Setting

The present research is a prospective observational study conducted in the Department of Radiodiagnosis at our institute over an 18-month period, from January 2024 to June 2025. The study included 42 patients aged 18 years and above who presented with peripheral pulmonary lesions (PPLs). Institutional Ethical Committee approval was obtained, and informed written consent was collected from all participants.

### Inclusion and Exclusion Criteria

**Figure 1:**
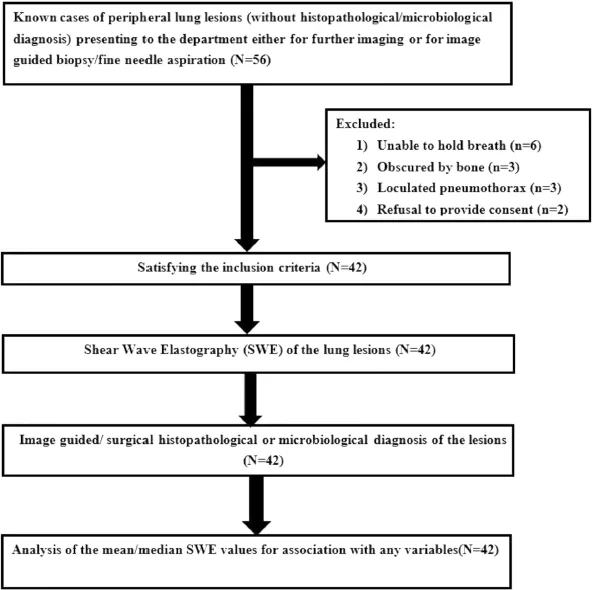
Flowchart design of the study

Patients were eligible for inclusion if they had peripheral pulmonary lesions measuring ≥20 mm, detected on chest radiograph or *Computed Tomography* (CT), and were referred from the Departments of Pulmonary Medicine or Internal Medicine for further evaluation. Exclusion criteria included patients younger than 18 years, those unable to hold their breath, lesions obscured by bone, presence of pleural effusion or pneumothorax, refusal to provide consent, or a lesion size of <20 mm. The study design is provided in Figure 1.

### Patient Enrollment and Clinical Assessment

Eligible patients underwent a clinical assessment and history-taking. Those meeting inclusion criteria were referred to the Department of Radiodiagnosis for imaging evaluation, which included both conventional ultrasonography and SWE. In patients requiring biopsy or further diagnostic intervention, imaging was performed before the procedure. Patients were then followed for histopathological or microbiological confirmation, wherever applicable.

### Ultrasound and Elastography Technique

Ultrasound examinations were performed by using the *Supersonic Mach 30* ultrasound system equipped with a C6-1X curvilinear convex probe operating at a frequency range of 1–5 MHz. Patients were examined in a supine or seated position based on lesion accessibility. B-mode greyscale ultrasound was first used to evaluate the lesion’s size, location, shape (regular or irregular), margin (smooth or rough), internal echogenicity, homogeneity, and the presence of air bronchograms.

Subsequently, SWE was performed. Patients were instructed to hold their breath during image acquisition in order to reduce motion artifacts. The SWE box was positioned over the lesion once a stable color signal was obtained. *Regions Of Interest* (ROIs) were placed on clearly visualized solid portions of the lesion, avoiding cystic, necrotic, or signal-void areas. The system automatically measured elasticity in kilopascals (kPa). The mean and median values for each lesion were calculated and used for further analysis.

### Imaging Data Analysis

All ultrasound and elastography images were independently analyzed by two radiologists blinded to the clinical and pathological outcomes. Lesions were characterized based on morphological features, echogenicity patterns, and the presence or absence of air bronchograms. These imaging findings were correlated with previous radiological studies (chest X-ray or CT), and histopathological or microbiological findings. Lesions considered benign were managed medically and monitored for resolution, while those with suspicious or persistent findings underwent image-guided biopsy.

### Statistical Analysis

Data were compiled by using Microsoft Excel and analyzed using SPSS version 23 (IBM Corp., Armonk, NY). Descriptive statistics were expressed as mean ± standard deviation and as frequencies and percentages for categorical variables. The Chi-square test or Fisher’s exact test were used to analyze associations between categorical variables. Continuous variables were compared using independent t-tests (for two groups) or one-way *ANOVA* (for more than two groups), followed by Tukey’s HSD for post-hoc analysis. For non-normally distributed data, the Mann–Whitney U or Kruskal–Wallis test were applied. Correlations between continuous variables were analyzed using Pearson’s or Spearman’s correlation coefficients, as appropriate. The diagnostic performance of SWE was evaluated in terms of sensitivity, specificity, positive predictive value (PPV), negative predictive value (NPV), and overall diagnostic accuracy. A *p*-value <0.05 was considered statistically significant.

## Results

### Demographic Profile

A total of 42 patients with peripheral pulmonary lesions were enrolled in the study. The mean age of the participants was 52.86 ± 13.76 years. Of the study population, 25 (59.5%) were male, and 17 (40.5%) were female, thus reflecting a slight male predominance. Cough (83.3%), followed by dyspnea (71.4%) were the most common symptoms. Demographic and clinical details are provided in Table 1.

**Table 1:** Demographic and clinical details of the study subjects

All parameters	Observation
**Age (Years)**	52.86 ± 13.76 (**Mean ± SD)**
**Gender**	
Male	25 (59.5%)
Female	17 (40.5%)
**Clinical features**	
Cough	35 (83.3%)
Dyspnea	30 (71.4%)
Weight loss	9 (21.4%)
Hemopytsis	11 (26.2%)
Chest pain/back pain	8 (19%)
Fever	3 (7.1%)
**Addiction history**	
Smoking	11 (26.2 %)
Alcoholism	7 (16.7%)
Smoking + alcoholism	10 (23.8 %)
Intravenous drug abuse	2 (4.8%)
No addiction	12 (28.5 %)
**HPE Impression**	
Benign	15 (35.7%)
Malignant	27 (64.3%)
**HPE Diagnosis**	
Adenocarcinoma	13 (31.0%)
Acute Inflammation	5 (11.9%)
Metastasis	5 (11.9%)
Chronic/Granulomatous Inflammation	3 (7.1%)
Fungal Infection	3 (7.1%)
Non-Small Cell Lung Carcinoma	3 (7.1%)
Tuberculosis	3 (7.1%)
Small Cell Carcinoma	2 (4.8%)
Squamous Cell Carcinoma	2 (4.8%)
Malignant Neoplasm (NOS)	1 (2.4%)
Malignant Thymoma	1 (2.4%)
Solitary Fibrous Tumor	1 (2.4%)

### Histopathological Diagnosis

Histopathological examination (HPE) revealed that 27 patients (64.3%) had malignant lesions, while 15 patients (35.7%) had benign lesions. The benign lesions included a range of inflammatory and infective etiologies such as granulomatous infections (including tuberculosis), organizing pneumonia, and fungal infections. Malignant lesions were diagnosed as squamous cell carcinoma, adenocarcinoma, small cell carcinoma, and metastatic tumors of extrapulmonary origin.

### Ultrasonographic Characteristics (Table 2)

On conventional ultrasound, the mean lesion diameter was 4.70 ± 1.76 cm. Twenty-four patients (57.1%) had lesions measuring less than 5 cm, whereas 18 patients (42.9%) had lesions 5 cm or larger. Lesions were located on the right side in 23 patients (54.8%) and on the left in 19 patients (45.2%). Regarding the shape, 23 lesions (54.8%) were irregular while 19 (45.2%) were regular. Most lesions (61.9%) had rough margins, and the remainder had smooth margins. Echogenicity assessment showed that 29 lesions (69.0%) were hypoechoic, while 13 lesions (31.0%) were hyperechoic. Air bronchograms were identified in 9 patients (21.4%) and were absent in 33 cases (78.6%).

**Table 2:** Sonographic features of the scanned subjects

Sonographic parameters	Observation
**Margin**	
Rough	26 (61.9%)
Smooth	16 (38.1%)
**Shape**	
Irregular	23 (54.8%)
Regular	19 (45.2%)
**Diameter (cm)**	4.70 ± 1.76 (**Mean ± SD)**
<5 cm	24 (57.1%)
≥5 cm	18 (42.9%)
**Location Side**	
Right	23 (54.8%)
Left	19 (45.2%)
**Air Bronchograms (Present)**	9 (21.4%)
**Echogenicity**	
Hypoechoic	29 (69.0%)
Hyperechoic	13 (31.0%)
**Mean Elastography Value (kPa)**	5.71 ± 2.69 (**Mean ± SD)**
**Median Elastography Value (kPa)**	5.74 ± 2.83 (**Mean ± SD)**

### Elastography Findings and Diagnostic Utility (Tables 3 and 4)

**Figure 2:**
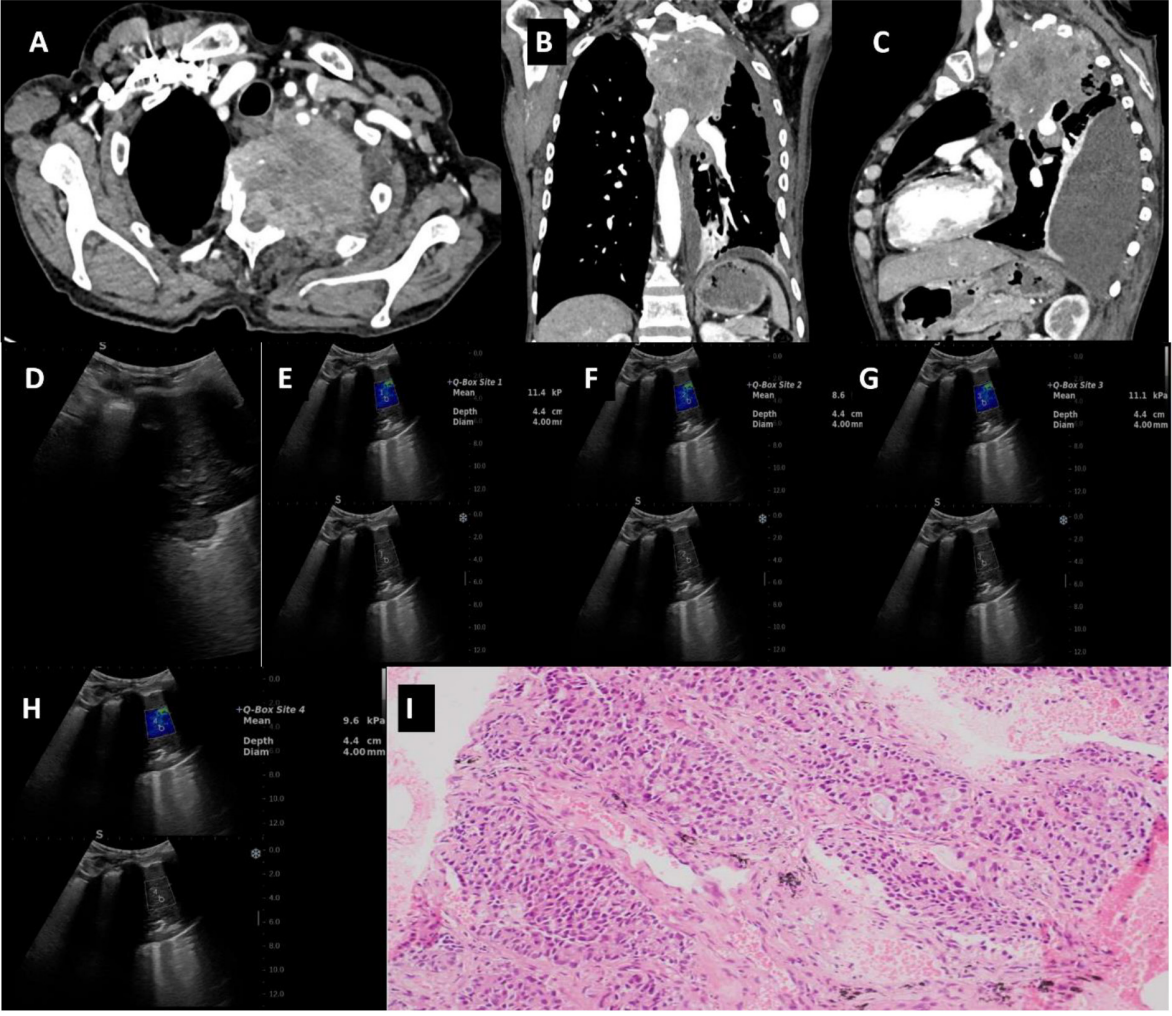
CECT chest mediastinal windows (A,B,C) in a 62-year-old male patient, former smoker, with complaints of cough, dyspnea and backache showing heterogeneously enhancing mass lesion in the left apical region with vertebral invasion. Greyscale USG (D) shows the sub-pleural hypoechoic lung lesion. SWE box was positioned on the lesion, and images were obtained. Four ROIs with a diameter of 3 mm were selected, which yielded values of 11.4 kPa, 8.6 kPa, 11.1 kPa and 9.6 kPa, respectively, with a mean ROIof 10.1 kPa. Hematoxylin and eosin (I) sections showing tumor cells arranged in sheets with a focal acinar pattern with intracytoplasmic mucin suggesting adenocarcinoma.

**Figure 3:**
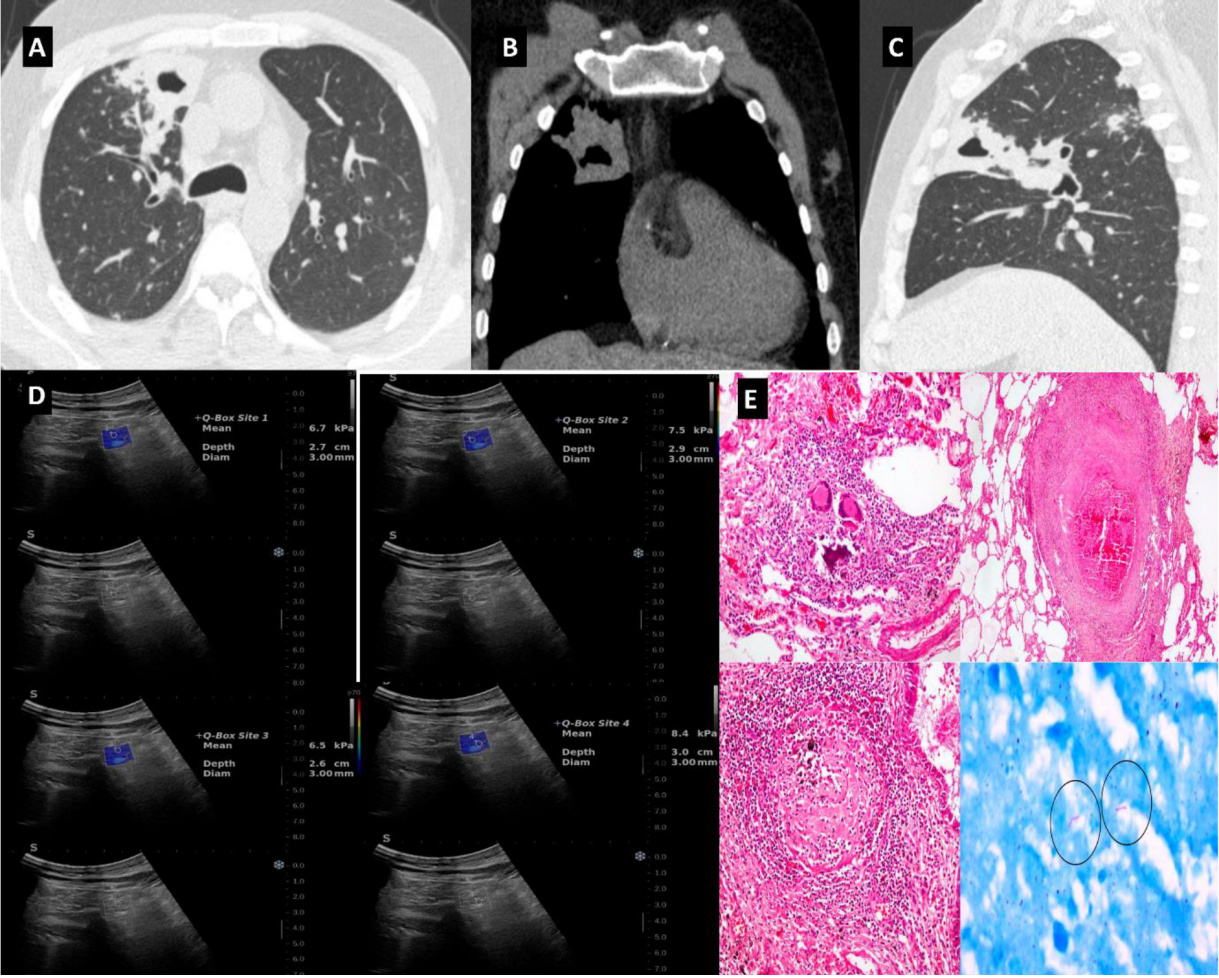
HRCT chest lung (A,C) and mediastinal (B) windows in a 43-year-old male patient with complaints of cough, dyspnea, weight loss & occasional hemoptysis showing an irregular and thick-walled cavitary lesion in the anterior segment of the right upper lobe. Four ROIs placed at the lesion on SWE yielded values of 6.7 kPa, 7.5 kPa, 6.5 kPa and 5.3 kPa, respectively, with a mean ROI of 6.5 kPa. Lung histopathology shows well-formed epithelioid cell granulomas with central necrosis and multinucleated giant cells on hematoxylin eosin staining with ZN staining highlighting acid fast bacilli (tuberculosis).

**Figure 4:**
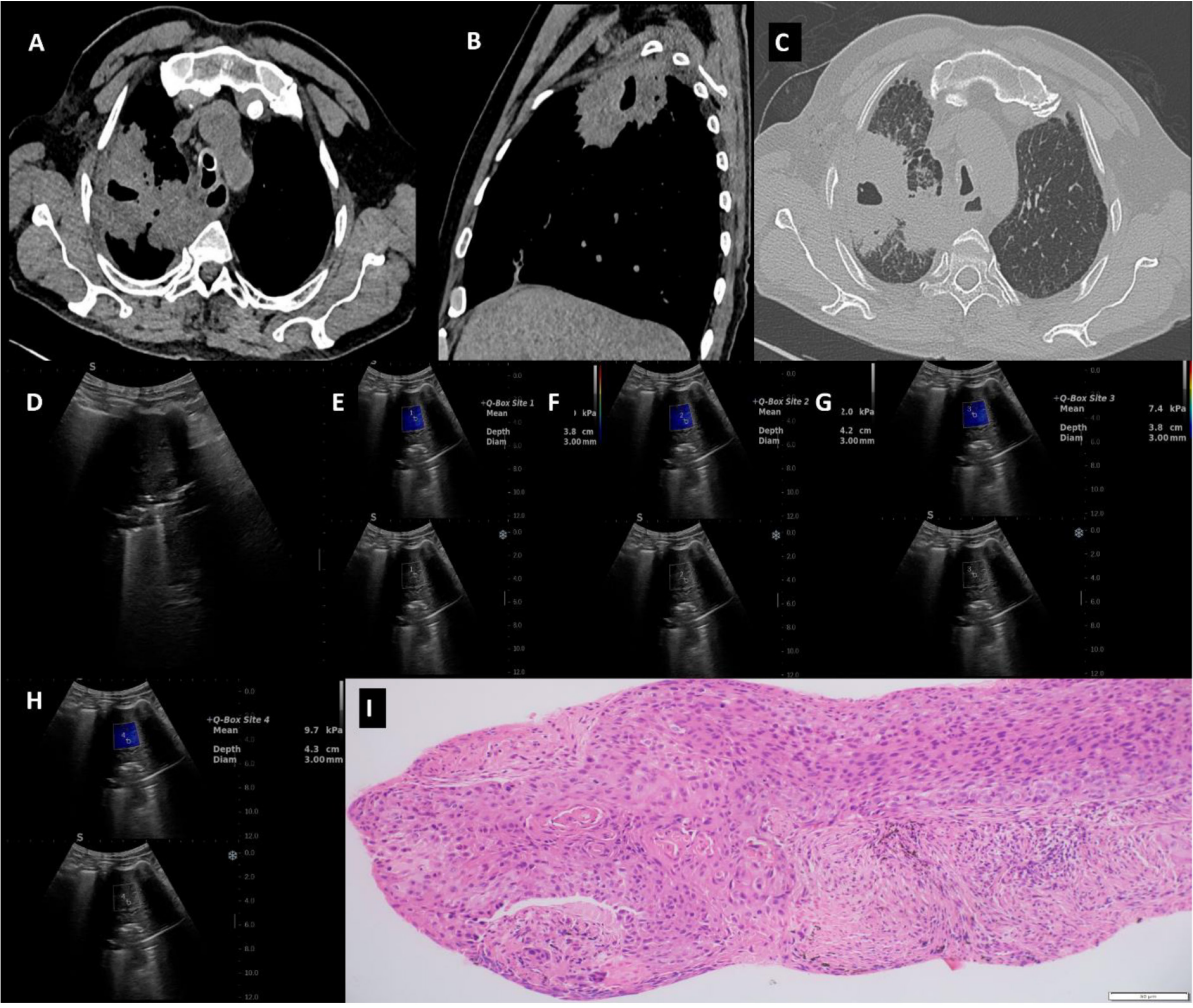
Mediastinal (A,B) and lung (C ) window sections of HRCT chest showing a spiculated cavitary lesion in the right lobe with elastography values of 10 kPa, 12 kPa, 7.4 kPa and 9.7 kPa. The mean came out to be 9.7 kPa. A biopsy revealed an invasive tumor arranged in nests, trabeculae and cords showing squamoid differentiation in the form of intercellular bridging and keratin pearl formation. Adjacent stroma shows a mild mixed inflammatory infiltrate. Features are of non-small cell carcinoma favoring squamous cell carcinoma – keratinizing type.

SWE was performed for all lesions, and the mean elastography value across the cohort was 5.71 ± 2.69 kPa. The mean SWE value in benign lesions was 3.72 ± 2.23 kPa, while in malignant lesions it was significantly higher at 6.82 ± 2.27 kPa. The distribution of SWE values was non-normal; hence, the Wilcoxon–Mann–Whitney U test was applied, revealing a statistically significant difference between benign and malignant groups (W = 61.000, *p* <0.001). The SWE values in benign lesions ranged from 1.52 to 9.72 kPa, whereas in malignant lesions they ranged from 2.85 to 10.6 kPa. A point-biserial correlation coefficient of 0.56 indicated a strong positive association between higher SWE values and malignancy. Figures 2–4 show few of our index cases.

**Table 3:** Association between Mean Elastography Value (kPa) and variable demographic, clinical and sonographic parameter

Parameters	Mean Elastography Value (kPa)	*p* value
Age (Years)***	Correlation Coefficient (r) = 0.34	0.028^1^
Age Group***		0.040^2^
20-40 Years	4.34 ± 2.84	
41-60 Years	5.20 ± 2.54	
>60 Years	6.92 ± 2.36	
**Gender**		0.258^3^
Male	6.12 ± 2.37	
Female	5.11 ± 3.07	
**Sonographic features**		
**Margin**		0.747^3^
Rough	5.60 ± 2.49	
Smooth	5.90 ± 3.06	
**Shape**		0.913^3^
Irregular	5.67 ± 2.53	
Regular	5.76 ± 2.94	
Diameter (cm)***	Correlation Coefficient (rho) = 0.53	<0.001^4^
Diameter Category***		0.002^5^
<5 cm	4.59 ± 2.31	
≥5 cm	7.21 ± 2.47	
**Location Side**		0.394^3^
Right	6.04 ± 2.79	
Left	5.32 ± 2.57	
Air Bronchograms***		0.014^5^
Present	3.69 ± 1.89	
Absent	6.26 ± 2.63	
**Echogenicity**		0.399^5^
Hypoechoic	5.52 ± 2.91	
Hyperechoic	6.15 ± 2.17	
**Median Elastography Value (kPa)**	Correlation Coefficient (r) = 0.99	<0.001^1^
**Diagnosis**		
**HPE Impression**		<0.001^5^
Benign	3.72 ± 2.23	
Malignant	6.82 ± 2.27	
**HPE Diagnosis**		0.004^2^
Adenocarcinoma	6.85 ± 1.84	
Acute Inflammation	2.52 ± 1.10	
Metastasis	7.22 ± 2.94	
Chronic/Granulomatous Inflammation	2.69 ± 0.51	
Fungal Infection	3.11 ± 1.07	
Non-Small Cell Lung Carcinoma	4.92 ± 1.37	
Tuberculosis	5.36 ± 1.78	
Small Cell Carcinoma	7.65 ± 2.47	
Squamous Cell Carcinoma	9.95 ± 0.92	
Malignant Neoplasm (NOS)	2.87 ± 0	
Malignant Thymoma	6.17 ± 0	
Solitary Fibrous Tumor	9.72 ± 0	

**Table 4:** Comparison of the diagnostic performance of the mean and median elastography values in differentiating benign vs. malignant lesions

Variable	Mean Elastography Value (kPa) (Cutoff: 4.8 by ROC)	Median Elastography Value (kPa) (Cutoff: 4.5 by ROC)
**Total Positives**	26 (61.9%)	26 (61.9%)
**True Positives**	23 (54.8%)	23 (54.8%)
**True Negatives**	12 (28.6%)	12 (28.6%)
**False Positives**	3 (7.1%)	3 (7.1%)
**False Negatives**	4 (9.5%)	4 (9.5%)
**Sensitivity**	85.2% (66-96)	85.2% (66-96)
**Specificity**	80.0% (52-96)	80.0% (52-96)
**PPV**	88.5% (70-98)	88.5% (70-98)
**NPV**	75.0% (48-93)	75.0% (48-93)
**Diagnostic Accuracy**	83.3% (69-93)	83.3% (69-93)
**AUROC**	0.849 (0.713 - 0.986)	0.825 (0.68 - 0.969)
**LR+**	4.26 (1.53-11.86)	4.26 (1.53-11.86)
**LR-**	0.19 (0.07-0.47)	0.19 (0.07-0.47)
**Yuden Index**	65.2	65.2
**Odds Ratio**	23 (4.41-119.96)	23 (4.41-119.96)
**Kappa**	0.64	0.64
***p*-Value**	<0.001	<0.001

### ROC Curve Analysis

*Receiver Operating Characteristic* (ROC) analysis demonstrated excellent diagnostic performance of SWE in distinguishing benign from malignant lesions. The area under the ROC curve (AUROC) for mean SWE values was 0.849 (95% CI: 0.713–0.986), which was statistically significant (*p* <0.001). A cutoff value of ≥4.8 kPa yielded a sensitivity of 85% and specificity of 80% for predicting malignancy. For median SWE values, the AUROC was 0.825 (95% CI: 0.680–0.969), with identical sensitivity and specificity at a cutoff of ≥4.5 kPa (*p* = 0.001).

### SWE Values across Pathologies

When SWE values were compared across different histopathological subtypes, significant differences were observed (χ^2^ = 27.523, *p* = 0.004), analyzed by using the Kruskal–Wallis test due to non-normal data distribution. Among malignant subtypes, squamous cell carcinoma exhibited the highest median SWE values. The strength of association between the SWE values and histological subtypes was statistically significant, though modest in magnitude (Kendall’s Tau = 0.12).

### Multivariable Analysis

In multivariable logistic regression, the mean elastography value (kPa) emerged as a significant independent predictor of malignancy in peripheral pulmonary lesions, with an odds ratio of 2.11 (95% CI: 1.18–4.87, *p* = 0.030). Although age showed a trend toward significance (OR = 1.09, 95% CI: 1.01–1.22, *p* = 0.055), other factors, such as the lesion shape, margin, echogenicity, diameter, and presence of air bronchograms were not statistically significant. The final model demonstrated strong discriminative performance (C-statistic = 0.909) and good calibration (Hosmer–Lemeshow *p* = 0.389), thereby confirming the diagnostic utility of shear wave elastography in differentiating malignant from benign lung lesions.

## Discussion

Peripheral pulmonary lesions encompass a diverse range of etiologies including benign inflammatory and infectious conditions (such as tuberculosis, fungal infections, and organizing pneumonia), as well as malignant neoplasms like squamous cell carcinoma, adenocarcinoma, small cell carcinoma, and metastatic tumors [8]. Accurate and timely differentiation between these entities is critical for patient management, especially as many malignant lesions can mimic benign pathologies radiologically and clinically.

While *Computed Tomography* (CT) remains the gold standard for imaging PPLs due to its excellent anatomical detail, its use is limited by ionizing radiation exposure and reduced specificity in differentiating the lesion etiology [8,9]. As a result, transthoracic ultrasound, particularly when enhanced with SWE, has gained traction as a complementary, radiation-free, and real-time diagnostic modality for evaluating subpleural lesions [10,11].

The mean SWE value across all lesions was 5.71 ± 2.69 kPa, with malignant lesions showing significantly higher stiffness (6.82 ± 2.27 kPa) than benign ones (3.72 ± 2.23 kPa, *p* <0.001). These results parallel earlier studies which also demonstrated significantly higher elastographic stiffness in malignant versus benign lung lesions [10–12]. Furthermore, the AUROC for mean SWE in our study was 0.849 (95% CI: 0.713–0.986), with a cutoff of ≥4.8 kPa yielding 85% sensitivity and 80% specificity, indicating high diagnostic performance. Although Mahmoud et al. reported similar AUROCs of 0.85 by using point SWE, their cutoff was higher at 23.3kPa [12].

When evaluating SWE values by the histological subtype, we found that squamous cell carcinoma exhibited the highest stiffness, which is a finding supported by studies showing increased stiffness in keratinized tumor subtypes [10,11]. Interestingly, tuberculosis and fungal infections also demonstrated elevated stiffness, occasionally exceeding malignancy cut-offs. This could be attributed to granulomatous inflammation, fibrosis, or cavitary wall thickening.

While an advanced age and smoking correlate with lung disease, our multivariable logistic regression identified mean SWE as an independent predictor of malignancy (*p* = 0.030, OR = 2.11), suggesting that its utility persists even when accounting for these variables. To minimize the impact of underlying lung fibrosis (often resulting from age or smoking), data collection was strictly confined to ROIs within the solid, pathological mass rather than the neighboring lung tissue. Furthermore, the increased stiffness in malignancy is primarily caused by dense cellularity and desmoplastic stroma rather than generalized lung aging. In our study, 26.2% were smokers, 7% were denoted by alcohol misuse, 23.8% were combined alcoholics and smokers, the diagnostic accuracy of SWE (83.3%) remained high across the cohort.

In terms of statistical modeling, mean SWE emerged as an independent predictor of malignancy, with an odds ratio of 2.11 (*p* = 0.030). Although other sonographic features (e.g., the lesion shape, air bronchograms, echogenicity) were evaluated, they did not reach statistical significance, which suggests that SWE may outperform the conventional sonographic features in differentiating lesion types.

Our study has several limitations. The relatively small sample size (n = 42) may limit the generalizability of the proposed stiffness cutoff (≥4.8 kPa) across broader and more diverse populations. SWE is inherently limited to the assessment of peripheral, pleura-contacting lesions and cannot reliably evaluate deep-seated masses or lesions obscured by bony structures; furthermore, the technique is dependent on patient cooperation, particularly adequate breath-holding so that to minimize motion artifacts. An overlap in stiffness values was also observed between malignant lesions and certain chronic inflammatory or infectious conditions, notably, tuberculosis, which can exhibit high stiffness and mimic malignancy. Importantly, the study by Quarto [13] found a substantial overlap between benign and malignant lesions which showed limited discriminatory utility of SWE. This heterogeneity is further emphasized by the considerably lower cutoff identified in our study compared with higher thresholds reported in the literature (23.3 kPa [12] and 65 kPa [14]), thereby highlighting the need for standardized SWE thresholds through larger, multicenter studies and uniform methodology.

## Conclusion

SWE is a promising noninvasive tool for differentiating between benign and malignant peripheral pulmonary lesions by providing quantitative tissue stiffness assessment. An elasticity cutoff of ≥4.5 Pa demonstrated good diagnostic performance for predicting malignancy. However, reported SWE cutoff values vary considerably across literature, thus highlighting the need for large, multicenter investigations in order to establish standardized thresholds and ensure consistent clinical application.
